# Localized Fluctuant Oscillatory Activity by Working Memory Load: A Simultaneous EEG-fMRI Study

**DOI:** 10.3389/fnbeh.2017.00215

**Published:** 2017-10-31

**Authors:** Xiaojie Zhao, Xiaoyun Li, Li Yao

**Affiliations:** College of Information Science and Technology, Beijing Normal University, Beijing, China

**Keywords:** memory load, oscillation, fMRI, joint independent component analysis, event-related spectral perturbation

## Abstract

Working memory (WM) is a resource-limited memory system for temporary storage and processing of brain information during the execution of cognitive tasks. Increased WM load will increase the amount and difficulty of memory information. Several studies have used electroencephalography (EEG) or functional magnetic resonance imaging (fMRI) to explore load-dependent cognition processing according to the time courses of electrophysiological activity or the spatial pattern of blood oxygen metabolic activity. However, the relationships between these two activities and the underlying neural mechanism are still unclear. In this study, using simultaneously collected EEG and fMRI data under an n-back verbal WM task, we modeled the spectral perturbation of EEG oscillation and fMRI activation through joint independent component analysis (JICA). Multi-channel oscillation features were also introduced into the JICA model for further analysis. The results showed that time-locked activity of theta and beta were modulated by memory load in the early stimuli evaluation stage, corresponding to the enhanced activation in the frontal and parietal lobe, which were involved in stimulus discrimination, information encoding and delay-period activity. In the late response selection stage, alpha and gamma activity changes dependent on the load correspond to enhanced activation in the areas of frontal, temporal and parietal lobes, which played important roles in attention, information extraction and memory retention. These findings suggest that the increases in memory load not only affect the intensity and time course of the EEG activities, but also lead to the enhanced activation of brain regions which plays different roles during different time periods of cognitive process of WM.

## Introduction

Working memory (WM) is a resource-limited memory system for temporary storage and processing of brain information during the execution of cognitive tasks, and plays an important role in complex cognitive activities (Baddeley, 1992). WM load refers to the ratio between the amount of memory information and the WM capacity. WM load that exceeds the limits of the WM capacity increases the complexity and difficulty of brain activities (Howard et al., 2003). Currently, electroencephalography (EEG) and functional magnetic resonance imaging (fMRI) are widely used to explore neural mechanism under different memory loads.

EEG studies on WM demonstrated that different oscillations react differently to an increase in memory load. More specifically, the power of theta, beta and gamma bands show an increasing trend (Jensen and Tesche, 2002; Deiber et al., 2007; Lundqvist et al., 2011) while the power of alpha presents a decreasing trend. Further studies reported that these changes in oscillatory activities have their unique temporal dynamics. Pesonen et al. (2007) used event-related spectral perturbation (ERSP) analysis to explore the time windows during which the event related synchronization (ERS) responses or event related desynchronization (ERD) of different bands were significantly related with an increasing memory load. Their results revealed that the time window of theta band (4–6 Hz) was at 0–1800 ms after stimulation, while that of the alpha band (8–12 Hz) was at 100–1600 ms. They also found that the beta band (14–23 Hz) exhibited ERS response under low memory load in 500–1800 ms post-stimulus and ERD response in 100 ms post-stimulus under high load (Pesonen et al., 2007). Based on these findings Palomäki et al. (2012) proved a steady increase of theta power after stimulation, while the power of alpha and beta (8–25 Hz) presented a continuous ERD response within 500–1800 ms under different memory loads. These studies suggested that the continuous theta ERS response depicted the mechanism underlying the information maintenance and extraction in addition to the close relationship between the alpha ERD response and cognitive process like memory requirements and information processing (Pesonen et al., 2007). Furthermore, changes in beta power are also associated with delay-period activity and stimulus discrimination (Tallon-Baudry et al., 1999; Varela et al., 2001). However, despite the available body of literature on the temporal dynamics of oscillations, no consensus has been reached regarding the range of time and frequency, or the trend of change.

Imaging studies on WM have demonstrated that the cingulate gyrus, inferior frontal gyrus (IFG), precentral gyrus, insula, middle frontal gyrus (MFG), cerebellum, inferior parietal lobule (IPL) and other brain regions play an important role in different phases of WM (Durgerian et al., 2001; Manoach et al., 2003; Kirschen et al., 2005). In a letter n-back experiment, Ragland et al. (2002) found load-dependent activation in the insula, IPL, MFG and some other brain regions, whereby a stronger activation was detected under higher memory loads. These brain areas play an important role in information maintenance and manipulation. Similar results were also observed in the superior parietal lobule (SPL) and cerebellum during the information encoding phase in a Sternberg memory task (Durgerian et al., 2001), and in the superior frontal gyrus (SFG) and MFG during the memory information maintenance phase (Jha and McCarthy, 2006). However, these previous findings were focused on the distribution of load-dependent fMRI activation and how they were affected by memory load, but not on the temporal dynamics of the changes in spatial activation.

The emergence of simultaneous EEG-fMRI provides a promising solution for this problem by integrating the high temporal resolution of EEG and the high spatial resolution of fMRI. This technique enabled researchers to gain deeper insights into the spatio-temporal characteristics of many classic cognitive functions, such as face recognition (Wirsich et al., 2014) and WM (Scheeringa et al., 2009; Michels et al., 2010; Ahmad et al., 2016; Herweg et al., 2016). Most of the EEG-fMRI studies on memory load were focused on the spatial distributions of specific event-related potential (ERP) components (D’Arcy et al., 2005; Sabri et al., 2014) and that of specific oscillatory activities (Scheeringa et al., 2009; Michels et al., 2010). Michels et al. (2010) used EEG-constrained fMRI analysis by introducing power value of an oscillation into the general linear model (GLM) as a covariate and identified the distribution patterns corresponding to theta and alpha activities under different loads. For theta power, these brain areas were mainly the medial prefrontal cortex and posterior parietal cortex (PPC). For alpha power, they were the dorsal lateral prefrontal cortex, PPC and some other brain regions. In one of our previous studies, we introduced ERP amplitudes into the GLM analysis and found that the P3 amplitude was suppressed under high memory load and that this suppression was correlated with activation in the middle occipital gyrus, insula, lingual gyrus and other brain regions (Zhang et al., 2017). Sabri et al. (2014) used joint independent component analysis (JICA) to study the association between ERP and fMRI in an auditory memory task. They demonstrated that the primary activation related to the N1 component occurred in the superior temporal gyrus and MFG, which was enhanced by higher memory loads. These studies proposed the spatial patterns of load-dependent ERP components and oscillations. However, whether the memory-load-dependent differences in temporal dynamics of oscillations are related to the differences in spatial activation distribution remains unclear.

In this study, we used JICA to explore the load-dependent relationship between temporal dynamics of oscillations and spatial activation distribution using simultaneous EEG-fMRI data from 13 healthy participants in a verbal n-back WM task. First, we used wavelet analysis to extract ERSP features from the EEG data of all channels under different memory loads. The ERSP features of an oscillation under the different memory loads were calculated and the ERSP difference was extracted using permutation test. The differential fMRI activation distribution was also obtained using GLM and *t*-test. Subsequently, the temporal ERSP difference and the spatial activation difference were modeled by JICA. Considering the correlation between adjacent channels, a multi-channel JICA (mJICA) model was constructed at the same time. Finally, correlation analysis was performed to investigate the relationship between the spatial activation of oscillations and behavioral performances. We suspected evidently load-effects oscillatory activities in the time phases in which concurrent stronger activation in specific regions of the frontal and parietal could be expected, and aimed to explore the temporal-spectral dynamics of electrophysiological activity and their spatial map of brain response to increasing memory load from a new perspective.

## Materials and Methods

### Participants and Experimental Procedure

Thirteen healthy right-handed participants (seven males and six females, mean age 22.9 ± 1.8 years) with no history of psychiatric or neurological disorders participated in the experiment after providing written statements of informed consent. The study was approved by the Institutional Review Board of the National Key Laboratory of Cognitive Neuroscience and Learning at Beijing Normal University. Simultaneous EEG-fMRI data were collected in the center for magnetic resonance imaging, brain cognitive science and technology at Beijing Normal University.

Our experiment used event-related design (Zhang et al., 2017) and the classic n-back WM paradigm with two memory load levels (*n* = 1, 3). The stimulations used were digits ranging from 0 to 9 which were presented in the center of a gray background. A black screen of 350 ms was presented to indicate the beginning of each trial. Subsequently, a fixation cross was presented at the center of the screen for 350 ms to direct the participants to focus on the target area. The stimulation then appeared at the same location for 500 ms and was followed by the cross appeared again for 2800 ms or 4800 ms, allowing the participants to make a quick key-press response. The participants were asked to compare the stimulus on the screen with the one that was displayed n-backwards. If a number was the same as the previous one (in 1-back task) or the third one backwards (in 3-back task), the participant was instructed to press the button under the right index finger. Otherwise, the participant was instructed to press a different button, the one under the right middle finger. The number of correct hits and the associated reaction time were recorded for each participant.

### EEG-fMRI Data Acquisition

The fMRI data were obtained using a 3T Siemens scanner at the MRI Center at Beijing Normal University using a standard echo-planar image (EPI) sequence (TR = 2000 ms, TE = 30 ms, matrix = 64 × 64, in-plane resolution = 3.12 × 3.13 mm^2^, slice thickness = 3.5 mm, slice gap = 0.8 mm, flip angle = 90°). Two-hundred images were collected at each stage. The whole brain was scanned and the interval scanning was carried out to obtain the axial images of the 33 layers parallel to the AC-PC line.

The EEG data were recorded simultaneously with 64 channels, using an MR-compatible amplifier (Brain Amp MR plus, Brain Products, Munich, Germany). The FCz channel was chosen as the reference electrode, and the sampling rate was set as 5000 Hz. Extra series resistors were required by all the electrodes to avoid saturation (5 kΩ for EEG and 15 kΩ for electrocardiography (ECG)). The scalp impedance was set below 20 kΩ.

### EEG Data Analysis

The preprocessing of the EEG data was conducted with Brain Vision Analyzer 2.0 (Brainproducts, Germany^1^). First, we removed the gradient and ballistocardiographic artifacts, filtered data from 1 Hz to 40 Hz with a band-pass filter, and down-sampled the data to 500 Hz. Subsequently, all EEG data were segmented into separate epochs based on the onset of each stimulus (200 ms pre-stimulus and 800 ms post-stimulus). All epochs were corrected to the baseline, and trials that met the following three criteria were identified as “bad” trials: (i) the trial was contaminated by excessive eye blink or motion artifacts; (ii) the amplitude exceeded ±150 μV; and (iii) the difference between the maximum and the minimum amplitude within 200 ms exceeded 200 μV.

Time-frequency (TF) analysis and statistical test were performed using EEGLAB 12.0.2.2b toolbox and TF representations were calculated using 2-cycle complex Morlet wavelets for both 1-back and 3-back epochs. The ERSP (Makeig, 1993) distributions for 12 representative electrode locations (F3, Fz, F4, P3, Pz, P4, C3, Cz, C4, O1, Oz, O2) were derived as a function of time (0–1000 ms from stimulus onset) and frequency (3–40 Hz, linear increase). A nonparametric permutation test was used to assess statistical differences between ERSP distributions (1-back vs. 3-back) with a permutation number of 1000 and an overall significance level of 0.01 (Maris and Oostenveld, 2007). The electrodes and oscillations with significant differences of ERSP between 1- and 3-back conditions were selected. The time course of ERSP in the 1–3 contrast was extracted from the same electrode for all participants.

### fMRI Data Analysis and ERSP-Activation Fusion

The fMRI data were preprocessed using SPM8^2^. The steps included slice timing, head motion correction, normalization to the Montreal Neurological Institute (MNI) space, reslicing into a resolution of 3 × 3 × 4 mm^3^, and spatial smoothing using a Gaussian kernel with a full-width at half maximum of 8 mm. The preprocessed fMRI data were modeled using GLM and yielded individual-level activation maps after parameter estimation and statistical test. One-sample *t*-test was performed on the individual activation maps of 1-back and 3-back condition respectively to generate group-level activation of each memory load and paired *t*-test was performed to generate group level activation difference between them.

The JICA (FITv2.0c^3^) was used to fuse EEG TF components and fMRI images, which assumes joint temporal and spatial independence constraints of these two modalities using the following generative model for the data: *X_EEG_* = *As_EEG_* and *X_fMRI_* = *As_fMRI_*. Here, *X*, *s* and *A* denote the observed signal, the independent sources, and a shared mixing matrix for two modalities data. In our study, for each contrasted oscillation, the fused dataset consisted of individual ERSP differences at the contrasted oscillations and individual contrasts of the activation map. The independence between spatial activation and temporal power perturbation was maximized using the fastICA algorithm, and the components that co-varied with both modalities were calculated and ranked by their contribution to the average ERSP time course. The number of independent joint components was determined using ICASSO (Himberg and Hyvarinen, 2003). The fMRI component was scaled to *z*-scores at threshold *z* > 2.0.

Apart from the single channel JICA (sJICA), the mJICA model was also used in our study that defined as below Swinnen et al. (2014).

$$\left[\begin{array}{cc} X_{EEG_{1}} & X_{fMRI}\\ X_{EEG_{2}} & X_{fMRI}\\ \ldots & \ldots \\ X_{EEG_{n}} & X_{fMRI} \end{array}\right]=A\cdot \left[s_{EEG}s_{fMRI}\right]$$

where, each row in *X*_EEGi_ represents the EEG time-courses at the *i*th channel for each participant.

### Correlation Analysis with Behavioral Performance

SPSS16.0 software was used to perform paired *t*-test on the participant’s behavioral data, including accuracy and reaction time. The fMRI component extracted by JICA was used as a mask to define individual regions of interest (ROI) as ROI_(oscillation)_. For each ROI_(oscillation)_, Pearson correlation of the mean blood-oxygen-level dependent (BOLD) value in 1-back with the behavioral data was conducted. In comparison, Pearson correlation of the mean BOLD value in 3-back with the behavioral data was also conducted to assess the influences of increasing load. The correlation coefficients between the activation difference and the behavioral difference according to memory load contrast were also calculated.

## Results

### Time-Frequency Activities from EEG

**Figure 1:**
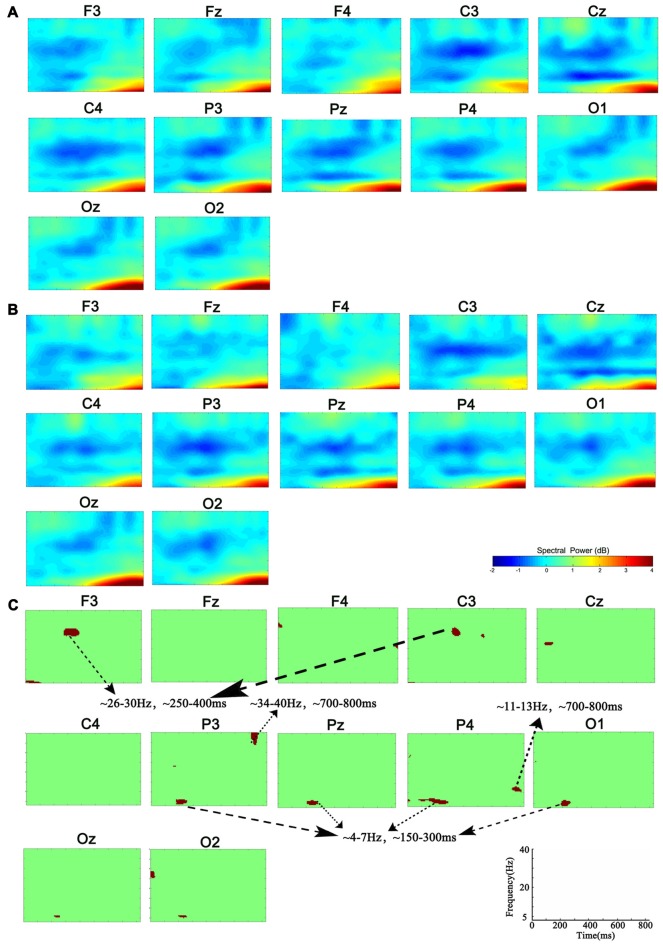
Group-level analysis of event-related spectral perturbation (ERSP). **(A)** ERSP distribution in 1-back condition. **(B)** ERSP distribution in 3-back condition. **(C)** Group difference of ERSP between 1- and 3-back conditions (*p* < 0.01). Significant difference of theta ERSP was observed in P3, P4, Pz and O1. Significant difference of beta ERSP was observed in F3 and C3. Significant difference of alpha ERSP was observed in P4. Significant difference of gamma ERSP was observed in P3.

**Figure 2:**
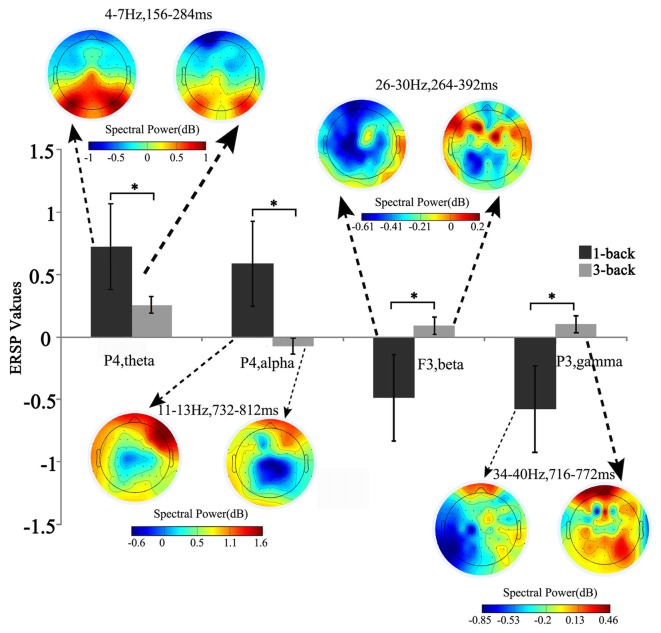
Brain topographic map and spectral perturbation differences between 1- and 3-back conditions at the specific electrode and time-frequency (TF) ranges. The power of theta increased at 150–300 ms to the power before the stimulus in all memory load conditions, and such increase slowed down under high memory load. For alpha oscillation in 700–800 ms post stimulus, the spectral perturbation showed an increasing tendency in low memory load and a decreasing tendency in high load. When compared with the baseline power, significant difference was detected in both beta (250–400 ms) and gamma (700–800 ms) power, which decreased under low memory load and increased under high load. *Paired *t*-test, *p* < 0.05.

Compared with the power of baseline, the spectral perturbation arose at different electrodes and TF range under different memory loads after stimulus onset, which showed ERS (positive ERSP value) or ERD response (negative ERSP value; Figure 1). For the specific electrode under specific TF range with significant difference, the oscillations presented various alterations in spectral perturbation across the whole brain when the memory load increased (Figure 2). The spectral perturbation of theta and alpha were anti-correlated with memory load, while that of beta and gamma were positively correlated. The time range with significant difference (namely time-SD), in which the ERSP showed apparent differences with various loads, was mainly observed at the early stage (before 400 ms) and the late stage (after 700 ms) after stimulus onset. The electrodes with notable changes in ERSP were selected as F3, C3, P3, Pz, P4 and O1 for the next fusion analysis.

### Spatial Activation of ERSP Time-Courses

**Figure 3:**
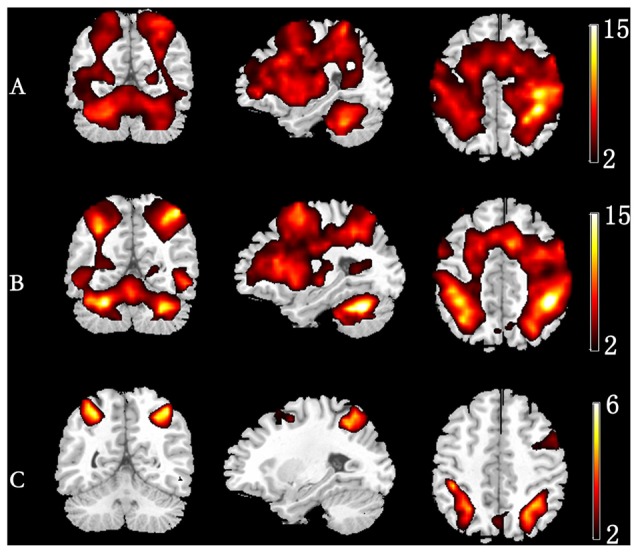
Group-level activation map in different memory load. **(A)** 1-back condition. **(B)** 3-back condition. **(C)** Group difference in activation (3-back vs. 1-back).

**Figure 4:**
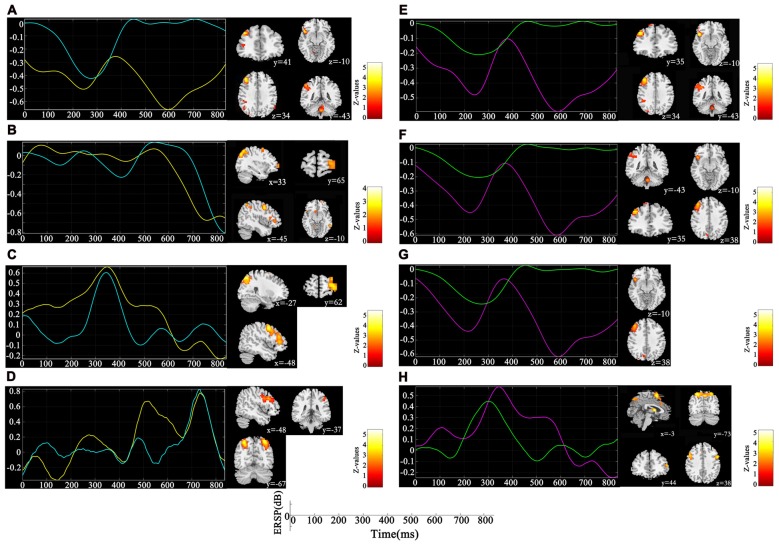
Components co-varied with both spectral perturbation difference in different oscillations and spatial activation difference detected by single channel joint independent component analysis (sJICA; left) and multi-channel JICA (mJICA; right). Both yellow line (left) and red line (right) represent the average time course of ERSP differences across participants and channels at the specific oscillation, and both blue line (left) and green line (right) represent the time independent component decomposed by JICA that most correlated with the average time course. **(A)** Theta oscillation of P4 channel. **(B)** Alpha oscillation of P4 channel. **(C)** Beta oscillation of F3 channel. **(D)** Gamma oscillation of P3 channel. **(E)** Theta oscillation of P4-Pz channels. **(F)** Theta oscillation of P4-Pz-O1 channels. **(G)** Theta oscillation of P4-Pz-P3-O1 channels. **(H)** Beta oscillation of F3-C3 channels.

Figure 3 depicts the significantly enhanced activation under high memory load, including the SFG, MFG, IFG, IPL and insula. For each participant, the difference of activation together with the difference of ERSP time-courses between 1-back and 3-back were put in the sJICA and mJICA model, respectively. The *z*-score maps of fMRI components generated by JICA were obtained after *z*-transform and were mapped to the MNI template. The number of independent components in the sJICA analysis was estimated to be 11 for theta-fMRI, 10 for alpha-fMRI, and nine for other-oscillations-fMRI. The number of independent components in mJICA was estimated to be nine. Among all the decomposed co-varied independent components, the temporal independent component of oscillation whose peaks changed significantly within the time-SD and its corresponding fMRI spatial independent component were selected (Figure 4).

### Correlation with Behavioral Performances

**Figure 5:**
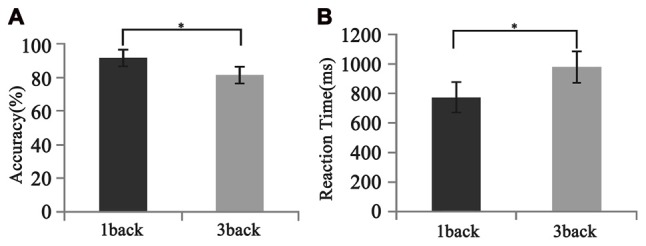
Different behavioral performances between 1- and 3-back conditions. **(A)** Accuracy. **(B)** Reaction time. Significant difference in behavioral performances was found between two loads. Error bar represents the standard error. *p < 0.05.

As is shown in Figure 5, all participants performed well in the behavioral test. The mean accuracy was 91.28 ± 8.36% and 81.11 ± 17.03% for 1-back and 3-back conditions, respectively. The mean reaction time was 774.7 ± 238.0 ms and 980.0 ± 357.7 ms for 1-back and 3-back conditions, respectively. The differences in both the accuracy and the reaction time between two loads were significant (*F*_(1,12)_ = 2.57, *p* < 0.025; *F*_(1,12)_ = −4.23, *p* < 0.001).

The correlation between the theta spatial activation and the accuracy in 1-back condition was more significant than that in 3-back condition. Meanwhile, the correlations between the alpha activation and the accuracy, between the beta activation and the reaction time, and between the gamma activation and the accuracy, were more significant in the 3-back condition than in the 1-back condition (Table 1).

**Table 1:** The correlation coefficients and their significant levels (*r*, *p*) between the mean blood-oxygen-level dependent (BOLD) of components that were linked with the event-related spectral perturbation (ERSP) of oscillation (ROI_(oscillation)_) and the behaviors.

ROI	1-back		3-back		Difference (1-back vs. 3-back)	
	Accuracy	RT	Accuracy	RT	Accuracy	RT
Left IFG_(θ)_	(0.53, 0.03)*	(−0.15, 0.32)	(0.43, 0.07)	(0.18, 0.26)	(−0.16, 0.31)	(0.21, 0.22)
Left MFG_(α)_	(0.44, 0.06)	(−0.06, 0.43)	(0.47, 0.05)*	(0.17, 0.29)	(−0.04, 0.44)	(0.15, 0.31)
Right SPL_(α)_	(0.32, 0.14)	(−0.21, 0.24)	(0.57, 0.02)*	(−0.18, 0.28)	(−0.22, 0.24)	(0.19, 0.27)
MFG_(β)_	(0.44, 0.07)	(−0.23, 0.23)	(0.29, 0.17)	(0.48, 0.05)*	(−0.01, 0.49)	(0.18, 0.27)
Right SPL_(γ)_	(0.08, 0.40)	(0.08, 0.40)	(0.49, 0.04)*	(−0.04, 0.45)	(−0.38, 0.10)	(0.32, 0.15)

RT, reaction time. *p < 0.05.

## Discussion

In this study, based on an n-back verbal WM task, we used JICA to bridge the differences of the EEG oscillatory activity and the differences of fMRI spatial activation, thus exploring the mechanism underlying the spatial-temporal processing of memory load. The results indicated that ERS response of theta was evoked in the early stage after stimulus onset by the increased memory load, consequently leading to enhanced activation in the SFG, IFG and MFG, which were involved in the memory delay-period activity. Besides, ERD response of alpha was evoked by high load in the late stage post-stimulus, leading to enhanced activation in the SFG, MFG and MTG, which were recruited in information extraction and memory retention. These results suggested that not only the covariant relations between the oscillatory activity and the activation of brain region, but also the roles of functional brain region in different temporal phases of the memory process.

In the early stage after stimulus onset, the changes in ERSP by loads occurred mainly in the neural activities of theta and beta (Figure 1). Unlike previous findings (Pesonen et al., 2007; Palomäki et al., 2012), our results indicated that the theta ERS response was negatively correlated with memory load. Previous studies reported that higher memory loads weakened the cognition control (Soutschek et al., 2013), while the ERS response of theta was associated with memory information encoding and retrieval (Jensen and Tesche, 2002). Based on this, the decreased theta ERS activity in the early stage suggested the manipulation of cognition control in the memory updating phase. For the beta activity, in line with previous study (Deiber et al., 2007), the ERSP and the ERS response increases during 250–400 ms post-stimulus under high load suggested that the beta activity was in charge of the completion of memory delay-period and memory encoding. In particular, the boost in beta power of delay-period may have been to promote stimulus recognition (Tallon-Baudry et al., 1999; Varela et al., 2001).

In contrast to the oscillatory activity in the early stage after stimulus onset, the late stage ERSP changes appeared mainly in the alpha and gamma activities. The spectral activity of alpha showed ERS response under low memory load but ERD response under high load. It has been proved that the alpha activity is engaged in information processing during the WM maintenance and is responsible for selective attention and semantic evaluation (Gomarus et al., 2006). Klimesch demonstrated that the alpha activity can actively inhibit the interference of irrelevant stimuli during cognitive tasks (Klimesch, 2012). Hence, the appearance of alpha ERD in the late post-stimulus stage under increased task difficulty may reflect the enhanced inhibition of irrelevant information during the stimulus evaluation. Similarly, due to the decreased task difficulty under low load, there may be less resource allocated to the inhibition of irrelevant stimuli. For the gamma activity, within 700–800 ms post-stimulus, the ERD response arose under low memory load, while the ERS response appeared under high load. Studies reported that continuous neural activities of gamma band are the physiological basis for the maintenance of information representation in WM tasks (Howard et al., 2003; Jokisch and Jensen, 2007), thus leading to the increase of gamma activities in memory information retention when the memory load increases (Howard et al., 2003).

The influence of WM load was reflected not only in different frequencies and different time windows, but also in the spatial distribution of brain activities. Higher WM demands led to stronger activation in the SFG, MFG, IFG, IPL and insula (Figure 3). After linking the spatial activation difference with the TF difference using sJICA (Figure 4A), the decreased theta activity in the early stimulus-response stage was co-varied with enhanced activation in SFG, IFG and IPL, which were involved in WM information encoding, recognition, and maintenance (Courtney et al., 1997; Durgerian et al., 2001; Jha and McCarthy, 2006). This suggested that the increased load may result in a weakened control over the cognitive activity, which will increase the difficulty of information encoding and maintenance. A stronger activation corresponding to the increased spectral perturbation of beta was observed in MFG, SPL and some other brain regions (Figure 4C). Previous studies have reported that activation in these brain areas reflected the memory load effect on the delay-period activity and information recognition (Varela et al., 2001; Manoach et al., 2003). In the late stimulus-response stage, the reduced alpha activity was co-varied with stronger activation in SFG, MFG and MTG (Figure 4B), which were mostly associated with memory information extraction and maintenance. This implies that, under high load, the increases in the target stimulus extraction and maintenance reflected the stronger suppression of irrelevant stimuli elicited by the alpha activity. Moreover, the stronger activation corresponding to the boost in gamma power was mainly observed in IPL and SPL, which may be in charge of the storage of linguistic information (Manoach et al., 2003; Figure 4D). These results manifest that the enhanced activation in some regions played various roles in different temporal phases of the memory process.

Compared with sJICA, more theta-related spatial activation was found in two-channel (Figure 4E) and three-channel (Figure 4F) with mJICA, such as the partial activation in the insula and the stronger activation in MFG. In the two-channel condition, an additional activation that corresponded to beta activity was detected in SFG and IFG. Besides, a stronger activation in SPL was also detected with mJICA (Figure 4H). However, when we further increased the number of channels, the JICA components became too scattered to form significant activations. One possible explanation may be that, with the increasing amount of channels, the reductions of correlation between the source signals resulted in the shrinking or even disappearance of the homologous space mode of these channel signals (Swinnen et al., 2014).

Consistent with previous findings, our behavioral data showed that with the increase of memory load, the reaction time increased significantly, while accuracy decreased significantly (Pesonen et al., 2007; Figure 5). However, no significant correlation was found between the activation difference fused by oscillatory activity and accuracy difference, or between the activation difference and reaction time difference (Table 1), implying that load-dependent changes in activation of oscillations was independent of behavioral changes. Compared with the 1-back condition, the correlation between the behavioral performance and the activation of alpha, beta and gamma significantly increased in the 3-back condition, which suggested that high memory demands increased the difficulty of the extraction, encoding, identification and maintenance of target stimulus information. As a result, the activation of corresponding brain areas in both the frontal and parietal cortex became stronger, the rate of correct responses decreased, and the reaction time became longer.

In summary, through the shared mixture matrix of JICA, a relationship between EEG temporal oscillatory activity and fMRI spatial activation based on the memory load contrasts was established. Limited by MR environment, EEG signals acquired with fMRI are vulnerable to several different types of artifacts, which make the signal-to-noise ratio (SNR) of EEG data particularly lower and make the data collection particularly difficult. However, EEG-fMRI simultaneous recording has the potential to enable the monitoring and modulation of brain activity over time using detailed brain spatial information. The present study, together with our previous studies (Zhang et al., 2017), aimed to reveal the possible brain mechanisms underlying the cognition processing of WM load using simultaneous EEG-fMRI technique from different point of view. In the future, the measures of oscillatory synchrony and BOLD-derived connectivity will be meaningful to further explore the deep relationship between activities of neurophysiology and neuroimaging, thus providing new insights on mental workload.

## Author Contributions

XZ and LY: conceived and designed the experiments. XL: analyzed the data. XL, XZ and LY: contributed reagents/materials/analysis tools. XZ and XL: wrote the manuscript.

## Conflict of Interest Statement

The authors declare that the research was conducted in the absence of any commercial or financial relationships that could be construed as a potential conflict of interest.

## Footnotes

^1^http://www.brainproducts.com

^2^http://www.fil.ion.ucl.ac.uk/spm

^3^http://mialab.mrn.org/software/fit/index.html
